# Association between Arachidonic Acid and the Risk of Schizophrenia: A Cross-National Study and Mendelian Randomization Analysis

**DOI:** 10.3390/nu15051195

**Published:** 2023-02-27

**Authors:** Yan Gao, Xiaowen Hu, Dandan Wang, Jie Jiang, Minghui Li, Ying Qing, Xuhan Yang, Juan Zhang, Yue Zhang, Chunling Wan

**Affiliations:** 1 Bio-X Institutes, Key Laboratory for the Genetics of Developmental and Neuropsychiatric Disorders (Ministry of Education), Shanghai Jiao Tong University, Shanghai 200030, China; 2 Department of Bioinformatics and Biostatistics, School of Life Sciences and Biotechnology, Shanghai Jiao Tong University, Shanghai 200240, China

**Keywords:** schizophrenia, arachidonic acid, long-chain polyunsaturated fatty acid, Mendelian randomization, cross-national study

## Abstract

Polyunsaturated fatty acids (PUFAs), especially long-chain PUFAs (LCPUFAs), are crucial for both the structural and functional integrity of cells. PUFAs have been reported to be insufficient in schizophrenia, and the resulting cell membrane impairments have been hypothesized as an etiological mechanism. However, the impact of PUFA deficiencies on the onset of schizophrenia remain uncertain. We investigated the associations between PUFAs consumption and schizophrenia incidence rates through correlational analyses and conducted Mendelian randomization analyses to reveal the causal effects. Using dietary PUFA consumption and national schizophrenia incidence rates in 24 countries, we found that incidence rates of schizophrenia were inversely correlated with arachidonic acid (AA) and ω-6 LCPUFA consumption (r_AA_ = −0.577, *p* < 0.01; r_ω-6 LCPUFA_ = −0.626, *p* < 0.001). Moreover, Mendelian randomization analyses revealed that genetically predicted AA and gamma-linolenic acid (GLA) were protective factors against schizophrenia (OR_AA_ = 0.986, OR_GLA_ = 0.148). In addition, no significant relationships were observed between schizophrenia and docosahexaenoic acid (DHA) or other ω-3 PUFAs. These findings show that the deficiencies of ω-6 LCPUFAs, especially AA, are associated with schizophrenia risk, which sheds novel insight into the etiology of schizophrenia and a promising diet supplementation for the prevention and treatment of schizophrenia.

## 1. Introduction

Schizophrenia is a complex psychotic disorder characterized by a bundle of neuropsychiatric manifestations, including cognitive impairment, apathy and social withdrawal [1]. The onset of schizophrenia typically occurs in young adulthood and can result in life-long impairment [1]. The median annual incidence rate of schizophrenia is 15.2 per 100,000 with substantial variations across countries and cultural groups [2]. Both genetic and environmental factors that impair neurodevelopment are considered risk factors for schizophrenia, including genetic predisposition provided by mutant neurodevelopmental genes and nutritional factors in prenatal life [1,3]. The indication of maternal malnutrition as a causative factor in schizophrenia was evidenced by higher incidence rates of schizophrenia among offspring during famines that occurred in the Netherlands during 1944–1945 [4] and in China during 1959–1961 [5].

An especially important nutrient group for the course of brain development is polyunsaturated fatty acids (PUFAs). PUFAs are essential fatty acids that are mainly obtained from the diet. The dietary intake of PUFAs varies widely due to geographic and cultural differences across countries [6]. Linoleic acid (LA, C18:2 ω-6) and alpha-linolenic acid (ALA, C18:3 ω-3) are the most abundant dietary PUFAs [6], and can be endogenously converted to long-chain PUFAs (LCPUFAs) to a limited degree. Arachidonic acids (AAs, C20:4 ω-6) and docosahexaenoic acid (DHA, C22:6 ω-3) are the major LCPUFAs in diets in most counties. LCPUFAs are an important nutrient group for brain development. In infancy and early childhood, both ω-6 and ω-3 LCPUFAs, especially AA and DHA, are critical for supporting brain growth and maturation [7]. Several studies on children have revealed improved performance in cognition and motor skills after LCPUFA supplementation [8,9,10], and LCPUFA deficiency can lead to psychotic symptoms in those with neurodevelopmental disorders [11]. Moreover, meta-analyses have shown that PUFA concentrations are insufficient in schizophrenia [12,13], and a similar tendency was found in individuals at an ultrahigh risk of developing psychosis [14].

Our study aims to investigate the associations between PUFAs and schizophrenia risk. Correlations between PUFA dietary consumption and national incidence rates of schizophrenia are evaluated from an epidemiological perspective, and Mendelian randomization (MR) analysis is conducted to address the causative relationships and evade residual confounding. We specifically hypothesize that lower LCPUFAs would be associated with an increased schizophrenia risk.

## 2. Materials and Methods

**Figure 1:**
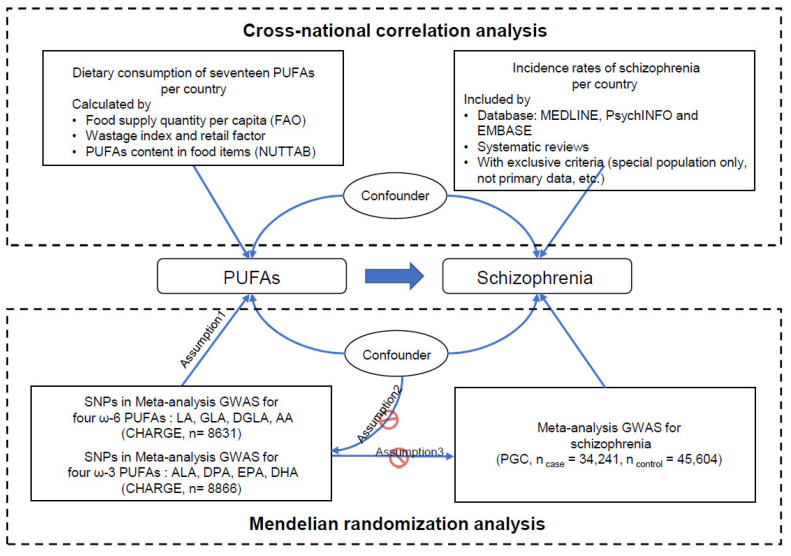
Schematic diagram of our study design. PUFA—polyunsaturated fatty acid; FAO—Food and Agriculture Organization of the United Nations; NUTTAB—Australian Food Composition Database (previously called NUTTAB); GWAS—genome-wide association study; SNP—single-nucleotide polymorphism; CHARGE—Cohorts for Heart and Aging Research in Genomic Epidemiology; LA—linoleic acid; GLA—γ-linolenic acid; DGLA—dihomo-γ-linolenic acid; AA—arachidonic acid; ALA—α-linolenic acid; EPA—eicosapentaenoic acid; DPA—docosapentaenoic acid; DHA—docosahexaenoic acid; and PGC—Psychiatry Genomics Consortium.

Our study consists of cross-national correlation analysis and MR analysis. A schematic diagram of our study design is shown as below (Figure 1).

### 2.1. Cross-National Correlation between PUFA Consumption and Schizophrenia Incidence Rates

#### 2.1.1. Data Acquisition on the Incidence Rates of Schizophrenia

The incidence rates of schizophrenia were obtained from published epidemiological studies. We included studies published between January 1975 and December 2021, which were identified through the search terms (incidence (Title/Abstract) AND schizophrenia (Title/Abstract)) applied in MEDLINE, PsychINFO and EMBASE, as well as the literature referred to in systematic reviews [2,15]. As the study design had a large impact on estimating the incidence rate of schizophrenia [16], the criteria for including studies in this analysis are listed as follows: Studies were included if they reported primary data on the incidence of schizophrenia according to the diagnostic criteria of the International Classification of Diseases (ICD) for schizophrenia. Studies within special populations only (e.g., twins, young adults) or within birth cohorts were excluded. When multiple studies on schizophrenia incidence in the same country were available, the study that reported the investigated population at risk as the total population or the one that sampled from the most recent period was preferred. Incidence was recorded as a rate per 100,000 population. Ethical approval was obtained in all original studies.

#### 2.1.2. Data Acquisition on the Dietary Consumption of PUFAs

Dietary consumption data of PUFAs were obtained from the Food and Agriculture Organization of the United Nations and the Australian Food Composition Database (previously called NUTTAB). The method of calculations followed the methodological process of establishing a global database of nutrients [17]. Briefly, food consumption data were obtained from the Food and Agriculture Organization, which annually compiles Supply and Utilization Accounts and provides internally consistent information about up to 394 food and agricultural commodities across nations. We extracted the available information about food supply quantity per capita (g/day) for countries with available incidence data (Table S1). To provide a more accurate estimate of the consumption of individual PUFAs, we introduced a wastage index to adjust for retail and consumption loss across countries [18] as well as the refuse factor to adjust for inedible parts [17]. Then, we matched these individual food items with the food items in the Australian Food Composition Database, a database that provides complete information on known dietary sources for PUFA intake. Finally, we added up the contributions of individual food items to each PUFA. Therefore, individual consumption of each PUFA within each country per year was calculated in aggregate. PUFA groups (total ω-3 PUFA, total ω-6 PUFA, total ω-3 LCPUFA, and total ω-6 LCPUFA) consumption was indirectly measured by summing up.

#### 2.1.3. Correlation Adjustment

A twenty-year lag interval was introduced to evaluate the causative relationships between PUFA intake in early life and schizophrenia risks in consideration of an adequate time span for the onset of psychiatric symptoms. Since PUFA consumption is correlated with economic development, we used the GDP per capita to account for the socioeconomic differences, paralleling previous studies [19,20,21,22].

#### 2.1.4. Statistical Analysis

In the cross-national association study, seventeen individual PUFAs and four summary PUFA groups were included for analyses. The analysis involved simple Pearson’s product moment correlation and partial correlation analyses adjusting for economic factors. These statistical procedures were conducted in SPSS 20.

### 2.2. Mendelian Randomization Analysis for PUFAs and Schizophrenia

To investigate the potential causal associations between PUFAs and schizophrenia, we applied a two-sample MR approach by using summary data from genome-wide association studies (GWASs). Ethical approval was obtained in all original studies.

#### 2.2.1. PUFA Exposure Data Acquisition

We obtained published association results for genetic instruments of ω-6 [23] and ω-3 PUFAs [24] from the Cohorts for Heart and Aging Research in Genomic Epidemiology (CHARGE) consortium, which are the most common datasets of ω-6 and ω-3 PUFA levels used for MR analysis [25,26,27,28]. The summary statistics were based on a meta-analysis of GWAS of individuals of European ancestry, which are 8631 European individuals for ω-6 PUFAs and 8866 European individuals for ω-3 PUFAs (Table 1). Participants for the meta-analysis were drawn from 5 cohort studies (Table S2), including the Atherosclerosis Risk in Communities Study (ARIC, n = 3269), the Cardiovascular Health Study (CHS, n = 2404), the Coronary Artery Risk Development in Young Adults Study (CARDIA, n = 1507), the Invecchiare in Chianti Study (InCHIANTI, n = 1075), and the Multi-Ethnic Study of Atherosclerosis (MESA, n = 707). In these studies, fatty acids were measured using gas chromatography technique. Levels of all individual fatty acids, including LA (average range, 19.96~24.78%), gamma-linolenic acid ((GLA) average range, 0.11~0.12%), dihomo-γ-LA ((DGLA) average range, 3.13~3.33%), AA (average range, 8.00~12.10%), ALA (average range, 0.14~0.44%), DHA (average range, 2.29~3.66%), eicosapentaenoic acid ((EPA) average range, 0.56~0.88%) and docosapentaenoic acid ((DPA) average range, 0.83~0.95%), were expressed as a percentage of total fatty acid (Table 1 and Table S2).

#### 2.2.2. Schizophrenia Outcome Data Acquisition

We obtained the summary statistics data on genetic associations for schizophrenia from Psychiatry Genomics Consortium (PGC) [29]. To mitigate population stratification bias, we extracted genetic data from the schizophrenia GWAS meta-analysis with the largest proportion of European population that included 36,989 cases and 113,075 controls, of which 34,241 sporadic cases and 45,604 ancestry-matched controls were used in the subsequent MR analysis (Table 1). To avoid bias due to overlapping sample sets in MR analyses, we checked the 49 individual participating studies of schizophrenia GWAS and found no overlap with the fatty acid GWASs. All genetic statistical data were downloaded from the PGC database (http://pgc.unc.edu, accessed on 28 January 2021).

#### 2.2.3. Univariable MR Analysis

In this MR analysis, four ω-6 PUFAs (LA, GLA, DGLA, and AA) and four ω-3 PUFAs (ALA, EPA, DHA, and DPA) were included. All included SNPs were significantly associated with PUFA levels at the genome-wide significance level (*p* < 5 × 10^−8^). Bias in effect estimates of MR analysis can be induced by correlation between SNPs. In order to minimize this bias, our genetic instruments used in each PUFA were limited to independent SNPs without linkage disequilibrium (R^2^ < 0.1). We calculated F statistics to assess the strength of each instrument. If F > 10, there is sufficient strength to avoid weak instrument bias in the MR analysis. The F statistics were computed by the formula F = (N-k-1) × R^2^/(1 − R^2^), in which R^2^ = 2 × MAF × (1 − MAF) × β^2^. N and k refer to the sample size and numbers of the instrument separately. All SNPs used in univariable MR analysis satisfied the MR criteria (F-statistic > 10) [30]. In addition, the instrumental SNPs are biologically relevant to PUFAs. The instruments of single PUFAs with adjustments for their precursors, including GLA, DGLA, and AA, were also obtained in ω-6 PUFAs GWAS from the CHARGE consortium [23].

We harmonized the summary-level data to ensure that the allele for each SNP corresponded between each PUFA and schizophrenia. In the main analysis, we used the fixed-effects inverse-variance-weighted (IVW) method to assess the causal effects between exposure and outcome. The odds ratio (OR) of schizophrenia was calculated per 1 SD increment in genetically predicted plasma fatty acid levels. We applied *p* < 0.05 as statistical significance criteria to classify SNPs as potentially influential. Two-sample MR analysis was implemented by using the “MendelianRandomization” package in R 4.0.

#### 2.2.4. Multivariable MR Analysis

We also conducted two-sample multivariable MR analysis to simultaneously estimate the direct effect of ω-3 and ω-6 PUFAs on schizophrenia in consideration of their nexus in metabolism. Considering the tight relationships between ω-3 and ω-6 PUFAs of sharing the same metabolic enzymes, multivariable MR analysis focused on estimating LA (18:2 ω-6) and ALA (18:3 ω-3) effects in comparison to each other, as well as AA (C20:4 ω-6) and EPA (C20:5 ω-3) effects in comparison to each other. Links regarding other PUFAs in equivalent positions in the synthesis pathway were not evaluated due to the lack of GWAS summary data from the same GAWS study. A total of eight SNPs related to ω-3 and ω-6 PUFAs were extracted from the CHARGE consortium. The final instrument set contained 5 SNPs due to linkage disequilibrium clumping. The schizophrenia outcome data were previously described. Multivariable MR was conducted using the R package “Mendelian Randomization”. The F-statistic was calculated to assess instrument strength, which was performed using the “MVMR” package.

#### 2.2.5. Pleiotropic Associations

We also considered potential biological and socioeconomic confounders that may affect the associations between PUFAs and schizophrenia risk. We explored the effect of some potential confounders such as C-reactive protein (CRP), educational attainment, and vitamin D levels [31,32,33]. A previous study found that instrumental variables of PUFAs were not causally related to education attainment and vitamin D [34], which illustrate that it was unlikely that PUFA instruments would affect the schizophrenia risk through these traits. Additionally, to address potential confounder CRP, we conducted linkage disequilibrium (LD) analyses [35] to investigate whether the inflammatory cytokine would affect the association between AA and schizophrenia risk. Genetic associations of CRP were obtained from a recent GWAS based on a large sample of >200,000 European individuals, which identified 58 genome-wide significant loci [36]. Four significant SNPs (rs10832027 and rs1582763 in chromosome 11, rs10521222 and rs1558902 in chromosome 16) in CRP GWAS are in the linkage distance to our instruments rs174547 and rs16966952 separately, which were assessed in the subsequent LD analyses. Furthermore, we searched PhenoScanner [37] for other potential pleiotropic associations of the instrumental variables with risk factors for schizophrenia.

## 3. Results

### 3.1. Cross-National Correlation between Dietary PUFA Intake and Schizophrenia Incidence Rates

Initially, 41 potentially related papers were collected based on the strict inclusion criteria described above to obtain national incidence rates of schizophrenia [38,39,40,41,42,43,44,45,46,47,48,49,50,51,52,53,54,55,56,57,58,59,60,61,62,63,64,65,66,67,68,69,70,71,72,73,74,75,76,77,78] (Table S3). Eight countries had more than one study reporting the incidence rate of schizophrenia. With further exclusions on studies decades ago or case findings based on the whole population rather than who was at an age known to be at risk, a unique incidence rate of each country was included for subsequent analysis (Table 2). The incidence rates of schizophrenia varied from 4.1 per 100,000 population in the United Kingdom to 58.5 per 100,000 population in India.

**Figure 2:**
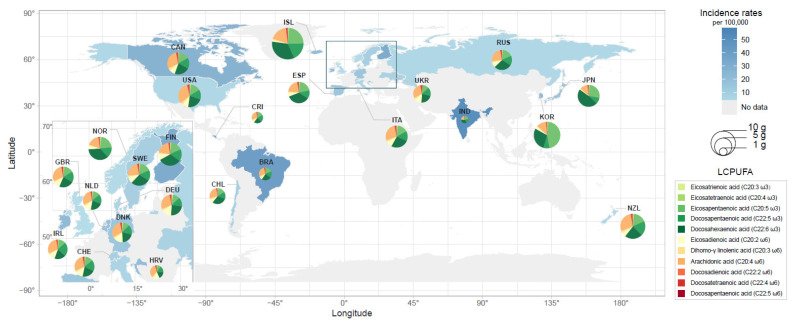
Incidence rates and estimated daily LCPUFA intakes for countries. Darker blue signifies higher incidence rates of schizophrenia and lighter blue signify lower rates. Grey tones indicate that the incidence rates of schizophrenia in these countries were not available in this study. The size of the pie chart is proportional to the dietary intake of total LCPUFAs. The NUTTAB contained 11 LCPUFAs in total, which green colors represent ω-3 LCPUFAs and orange colors represent ω-6 LCPUFAs. The area outlined by the dark-blue box zoomed into the lower left corner of this figure. BRA—Brazil; CAN—Canada; CRI—Costa Rica; HRV—Croatia; DNK—Denmark; FIN—Finland; DEU—Germany; ISL—Iceland; IND—India; IRL—Ireland; ITA—Italy; JPN—Japan; NLD—Netherlands; NZL—New Zealand; NOR—Norway; RUS—Russian Federation; ESP—Spain; SWE—Sweden; CHE—Switzerland; KOR—the Republic of Korea; UKR—Ukraine; GBR—United Kingdom; and USA—United States.

Eating habits vary across different countries and cultures, leading to a global difference in the major dietary PUFA categories and a wide range of PUFA intake. The NUTTAB included 17 dietary PUFAs, and the scales of the dietary PUFAs differed by two orders of magnitude; on average, LA accounted for 88.3% of the total PUFA intake, whereas adrenic acid (AdrA, C22:4 ω-6) accounted for only 0.08%. AA and DHA were the major individual LCPUFAs among most countries (Figure 2). PUFA compositions of daily intake across countries and a potential confounder, GDP per capita, are displayed in Table 2.

**Figure 3:**
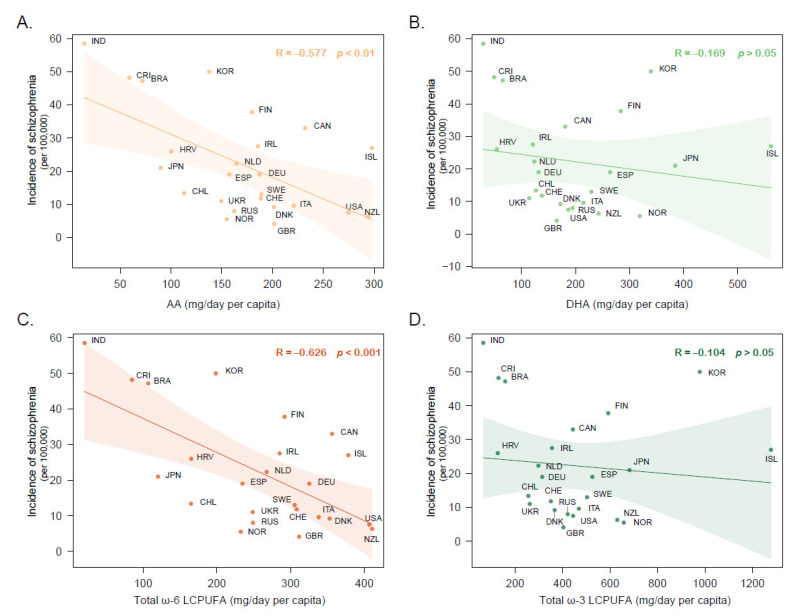
Correlations between schizophrenia incidence rates and (**A**) AA, (**B**) DHA, (**C**) total ω-6 LCPUFA, and (**D**) total ω-3 LCPUFA. Correlations described the relationship differences for AA (r_AA_ = −0.577, *p* < 0.01), DHA (r_DHA_ = −0.169, *p* > 0.05), total ω-6 LCPUFA (r_ω-6 LCPUFA_ = −0.626, *p* < 0.001), and total ω-3 LCPUFA (r_ω-3 LCPUFA_ = −0.104, *p* > 0.05).

Correlation analyses showed that a higher consumption of AA was correlated with a lower schizophrenia incidence rate (r_AA_ = −0.577, *p* < 0.01) (Figure 3A). Moreover, its precursor (eicosadienoic acid, C20:2 ω-6) and product (AdrA) were related to schizophrenia incidence rates in similar relationships. Dietary consumption of other individual PUFAs, including DHA, did not show statistically significant correlations (Figure 3B). Given the potential socioeconomic effects on the consumption of food, we introduced the GDP per capita across different countries to adjust economic levels by partial correlation analysis. As a result, AA consumption remained inversely correlated with the incidence rates of schizophrenia.

We further investigated schizophrenia incidence rates against different PUFA families. Similarly, the results showed that higher ω-6 LCPUFA consumption was correlated with a lower incidence rate of schizophrenia (r_ω-6 LCPUFA_ = −0.626, *p* < 0.001) (Figure 3C), whereas no significant relationship was observed between ω-3 LCPUFA consumption and schizophrenia incidence rates (Figure 3D). In addition to LCPUFAs, an association analysis between total ω-6 PUFA consumption and schizophrenia was conducted and found no significant relationship. Total ω-3 PUFA consumption showed a weak negative association with schizophrenia incidence rates, which was no longer significant after adjustments for economic factors. In addition, there were no correlational relationships between the incidence rates of bipolar disorder or depressive disorder and dietary PUFAs within the limited available dataset [79,80,81,82] (Table S4).

Taken together, these results suggest that the high intake of ω-6 LCPUFAs, especially AA, would decrease the risk of schizophrenia onset.

### 3.2. Mendelian Randomization Analysis of PUFA and Schizophrenia

**Figure 4:**
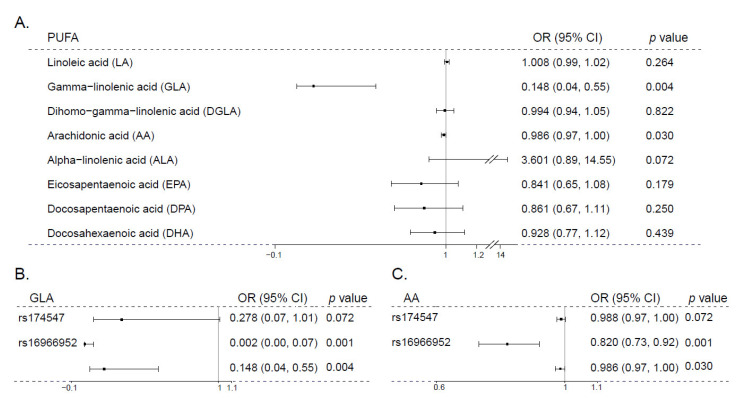
Associations of genetically predicted PUFA levels with schizophrenia. (**A**) This figure shows the Mendelian randomization inverse-variance-weighted (IVW) results for the relationship between the eight PUFAs and the risk for schizophrenia. (**B**,**C**) Forest plots of the individual SNP effects for genetically predicted GLA and AA levels on schizophrenia risk. These figures illustrate the contribution of individual instrumental variables used for the association between (**B**) GLA, (**C**) AA, and schizophrenia risk. PUFA—polyunsaturated fatty acid; OR—odds ratio per SD; and 95% CI—95% confidence interval. The middle line represents the null (OR = 1.000), and the error bars represent 95% CI.

To further address the causal relationship between PUFAs and schizophrenia, we performed MR analyses. The variants used as genetic instrumental variables of the eight different PUFAs are listed in Table 3. These SNPs regarding each PUFA were independent without linkage disequilibrium and were not directly associated with schizophrenia at the genome-wide significance threshold. For all instruments related to each PUFA, F-statistics > 10, suggesting that the analyses were unlikely to be affected by weak instrument bias (Table 3). The results of the univariable MR analyses were based on the inverse-variance-weighted method. For ω-6 PUFAs, genetic predisposition to higher levels of AA or GLA was significantly associated with lower schizophrenia risk (OR_AA_ = 0.986, *p* = 0.030; OR_GLA_ = 0.148, *p* = 0.004), whereas ω-3 LCPUFAs showed only protective tendencies without statistical significance (Figure 4A). In contrast, the precursors of LCPUFAs, LA and ALA, showed the opposite effects on schizophrenia (OR_LA_ = 1.008, *p* = 0.264; OR_ALA_ = 3.601, *p* = 0.072) (Figure 4A). Single-SNP analyses between AA and schizophrenia showed that the direction of the causal relationship driven by rs174547 was the same as the direction driven by rs16966952. The results of single-SNP analyses between GLA and schizophrenia were similar (Figure 4B,C).

These associations were driven by the SNP rs174547 in *FADS1* and rs16966952 in *NTAN1/PDXDC1*. These genes are strongly connected with fatty acid metabolism. FADS1 is involved in the desaturation of LA and ALA, contributing to the biosynthesis of LCPUFAs such as AA and DHA. PDXDC1 expression level appeared to be changed in mice fed with a high-fat diet.

The pleiotropy assessment found that rs174547 was most strongly associated with AA and other multiple metabolic traits that are closely related to PUFAs, such as lipid metabolism phenotypes. The SNP rs16966952 was predominantly associated with DGLA and PUFA-related metabolic traits (Table S5). Given the instrumental variables related, most of the traits are downstream of the main exposure of interest. These metabolic relationships are considered as vertical pleiotropy and have no effects on the consequence of MR analysis. Other traits include pulse rate, peripheral blood cell count, height, asthma, hair, or balding pattern, none of which are considered as potential risk factors for schizophrenia. Moreover, neither of these SNPs was directly related to the other risk factors for schizophrenia at the genome-wide significance threshold (Table S5). As for some potential confounders such as CRP and educational attainment and vitamin D levels, there are some evidences to manifest AA-related SNPs would not affect the schizophrenia risk through these traits. Our LD analysis results show that all of the significant SNPs of CRP were independent with our instruments rs174547 and rs16966952 (R^2^ < 0.1), which implied that CRP is not genetically related to AA (Figure S1). A previous study reported that AA is not causally related to educational attainment and vitamin D [34]. These results suggest it is unlikely that the genetic instruments of AA and GLA affect schizophrenia via these confounders.

Considering the close relationships between PUFAs, we applied further MR analyses. Given the sharing of the elongating and desaturating enzymes by ω-6 and ω-3 PUFAs, multivariable MR analyses were applied to estimate the direct effects of one fatty acid in consideration of another. In the associations between schizophrenia and two PUFAs, AA and EPA, which were assessed together, there is evidence for a causal effect of AA as a protective factor of schizophrenia (OR_AA_ = 0.981, *p* = 0.009), whereas no statistically significant evidence was found with EPA (Table 4). In the associations between schizophrenia and LA and ALA, which were assessed together, effect estimates for LA and ALA aligned well with the univariable MR estimates (OR_LA_ = 1.005, *p* = 0.805; OR_ALA_ = 3.747, *p* = 0.512) (Table 4). Given the metabolic pathway in each PUFA group, further univariable MR analyses were applied to estimate the effect of a single PUFA within consideration of its preceding fatty acid. In this MR analysis, controlling for precursor PUFA, low schizophrenia risk was consistently observed with higher levels of AA and GLA (OR_AA_ = 0.986, *p* = 0.021; OR_GLA_ =0.240, *p* = 0.002) (Table S6).

Overall, the MR analyses indicate that AA and GLA are protective factors against schizophrenia. Consistent with our cross-national correlation results, insufficient AA may be a potential causal factor leading to the onset of schizophrenia.

## 4. Discussion

In this study, we aimed to investigate the relationships between PUFAs and schizophrenia risk using epidemiological methodology and Mendelian randomization analysis. Our results showed that ω-6 LCPUFAs, especially AA, are potential protective factors against schizophrenia. A higher intake of AA and ω-6 LCPUFAs, according to measures of food consumption, was significantly correlated with a lower incidence rate of schizophrenia across countries. Consistently, a genetically predicted low level of AA was revealed to increase schizophrenia risk by the MR analysis. In contrast, ω-3 LCPUFAs, including DHA and EPA, showed only a slight tendency to be protective against schizophrenia risk, which was an effect without statistical significance.

Both ω-3 and ω-6 LCPUFAs are rich in seafood and animal food. Previous studies have focused on the correlational relationships between the prevalence of affective disorders and seafood consumption [83,84] and found that lower seafood consumption was robustly associated with higher prevalence rates. Our study focused on the relationships between PUFAs and schizophrenia, and indicates that a high intake of ω-6 LCPUFAs, especially AA, was associated with a low risk of schizophrenia. Careful consideration was given to potential confounding factors. The relationship between each PUFA and schizophrenia was adjusted for based on country socioeconomic levels assessed by GDP per capita. Correspondingly, the results of our two-sample MR study, which is less prone to confounding than cross-national correlation studies, showed that AA was a protective factor against schizophrenia risk. Our MR results were based on large GWASs and the same race, which minimized the possibility of population stratification bias. The genes involved in the MR analysis, the *FADS1* gene in LCPUFA synthesis, was reported to influence IQ performance, illustrating the crucial effects of PUFAs on early brain development [85]. NTAN1 is an *N*-terminal asparagine amidase that is related to social behavior and memory [86]. The results conducted by the MR analyses reveal that these variants may not directly lead to disease but instead affect schizophrenia risk by altering LCPUFAs, especially AA, and their subsequent traits. Therefore, these results both indicate that AA may play an important protective role against schizophrenia onset.

Although both ω-6 and ω-3 LCPUFAs are critical in brain function and development, our results show that ω-6 LCPUFAs, but not ω-3 LCPUFAs, function as protective factors against developing schizophrenia. Our findings provide supportive evidence for the hypothesis that the onset of schizophrenia might be determined by insufficient AA, a major ω-6 LCPUFA in the brain; this is released for the production of its eicosanoid metabolites to support adequate signal transduction [87], and when present at abnormal levels, mental activities due to the structural enrichment of AA and its vital functions in the brain will be disrupted.

AA deficiency impairs normal brain function [88] and contributes to schizophrenia-like phenotypes [89], and supplementation with AA can alleviate psychotic manifestations to some extent [9,10,90,91,92,93]. Lee et al. found that *Lpiat1*^−/−^ mice, a model of AA deficiency in phosphatidylinositol synthesis, died within a month after birth and showed atrophy of the cerebral cortex and hippocampus [88]. M. Maekawa et al. found that gestational and early postnatal dietary deprivation of AA in mouse offspring elicited schizophrenia-like phenotypes in adulthood [89]. Meanwhile, M. Maekawa et al. [90] found that additional AA in breastmilk increases the total number of neurons in not only normal infant rats but also schizophrenia-like infant rats with information-processing problems, whereas few effects of DHA administration were observed. Other findings showed that high levels of AA helped improve cognitive development in children [9,10] and alleviate cognitive dysfunction in elderly individuals [91,92,93]. A recent study further confirmed this finding by observing that higher ω-6 LCPUFA levels in childhood, but not ω-3 LCPUFA, reduced the risk of psychotic experiences or psychotic disorder in adulthood [11]. These findings suggested that AA plays an essential role in neurodevelopment, which manifests as both a disruption in cognitive performance and psychotic symptoms during the onset of schizophrenia. Our findings provide supportive evidence from an epidemiological perspective and an MR-analysis-based causal inference.

In contrast to AA and ω-6 LCPUFAs, we found that ω-3 LCPUFAs were not significantly related to schizophrenia risk. Several cross-national studies reported strong relationships between fish consumption and mood disorder [83,84,94], which indicates that ω-3 LCPUFAs may have an impact on affective disorders. In contrast, there were no associations between seafood consumption and schizophrenia prevalence [84]. Furthermore, M. Maekawa et al. found that mice supplemented with DHA showed better behaviors in sustaining motivation or the propensity to respond, while mice supplemented with AA showed better performance in a cognitive processing task [89]. Meta-analyses have indicated that ω-3 LCPUFA supplementation may have beneficial effects on affective symptoms in major depressive disorder [95] and bipolar depression [96], whereas it failed to reveal therapeutic benefits on psychotic symptoms in schizophrenia [97]. Taken together, these findings also indicated that ω-3 LCPUFA showed limited influence on the onset of schizophrenia.

## 5. Conclusions

In conclusion, our findings, combining the results of diet-dependent epidemiological analyses and gene-dependent MR analyses, show that AA and ω-6 LCPUFAs are protective factors against schizophrenia risk. These results imply diet enrichment might help prevent the onset of schizophrenia. Studies on AA supplementation for individuals with ultra-high risk for psychosis would help further characterize the nature of the AA and schizophrenia associations.

## Figures and Tables

**Table 1:** Information of data on PUFAs and schizophrenia of MR analysis.

Phenotype	Sample Size	Ancestry	Women%	PUFA Concentration, % of Total Fatty Acid
**ω-6 PUFAs ^a^**	8631	Europe	51~62%	LA (19.96~24.78%), GLA (0.11~0.12%), DGLA (3.13~3.33%), AA (8.00~12.10%)
**ω-3 PUFAs ^b^**	8866	Europe	51~61%	ALA (0.14~0.44%), DHA (2.29~3.66%), EPA (0.56~0.88%), DPA (0.83~0.95%)
**Schizophrenia ^c^**	34,241 cases, 45,604 controls	93.5% European 6.5% East Asian	8~73%	Not applicable

^a^ Summary statistic for GWAS of ω-6 PUFA from “Genome-wide association study of plasma N6 polyunsaturated fatty acids within the cohorts for heart and aging research in genomic epidemiology consortium” [23]. ^b^ Summary statistics for GWAS of ω-3 PUFA from “Genetic loci associated with plasma phospholipid *n*-3 fatty acids: a meta-analysis of genome-wide association studies from the CHARGE Consortium” [24]. ^c^ Summary statistics for GWAS of schizophrenia from “Biological insights from 108 schizophrenia-associated genetic loci” [29]. PUFA—polyunsaturated fatty acid; LA—linoleic acid; GLA—γ-linolenic acid; DGLA—dihomo-γ-linolenic acid; AA—arachidonic acid; ALA—α-linolenic acid; EPA—eicosapentaenoic acid; DPA—docosapentaenoic acid; and DHA—docosahexaenoic acid.

**Table 2:** The incidence rates of schizophrenia, PUFA consumption, and economic development across different countries.

Country Code	Country	Incidence of Schizophrenia			PUFA Consumption ^a^				Economic Variable
		Coverage	Period	Incidence (Per 100,000)	AA (mg/Day)	DHA (mg/Day)	ω-6 LCPUFA (mg/Day)	ω-3 LCPUFA (mg/Day)	GDP Per Capita ^b^
BRA	Brazil	Subnational	1965–1994	47.2 [38]	71.97	65.35	106.99	158.03	431.3
CAN	Canada	Subnational	1995–1998	33.0 [39]	231.99	180.74	356.23	443.35	8590.8
CHL	Chile	National	2004–2017	13.4 [40]	113.12	126.64	164.82	255.50	3076.6
CRI	Costa Rica	National	1979–1981	48.2 [41]	59.00	49.29	84.85	129.73	355.0
HRV	Croatia	National	1980–1985	26.0 [42]	100.14	54.09	165.14	126.67	4852.2
DNK	Denmark	National	1995–2008	9.2 [43]	201.39	172.04	352.87	366.56	13,333.0
FIN	Finland	National	2003	37.8 [44]	179.83	283.62	291.77	592.44	10,497.5
DEU	Germany	National	1987–1989	19.0 [45]	187.32	131.59	325.37	314.24	2761.2
ISL	Iceland	National	1967	27.0 [46]	297.87	561.37	378.27	1278.49	1418.1
IND	India	Subnational	1987–1988	58.5 [47]	14.70	29.26	20.41	65.78	98.1
IRL	Ireland	Subnational	1974–1977	27.5 [48]	185.71	120.95	285.16	355.25	739.3
ITA	Italy	Subnational	2008–2011	9.6 [49]	220.95	214.39	338.11	468.64	18,728.4
JPN	Japan	Subnational	1979–1980	21.0 [50]	89.70	384.11	120.07	682.04	563.6
NLD	Netherlands	Subnational	1981–1997	22.3 [51]	164.43	123.68	267.52	297.9	3662.2
NZL	New Zealand	National	2014	6.3 [52]	294.28	242.54	410.68	630.07	17,400.4
NOR	Norway	Subnational	1982–1983	5.5 [53]	154.97	318.68	232.38	658.06	1721.4
KOR	Republic of Korea	National	2008–2017	50.0 [54]	137.70	339.12	198.58	978.261	9057.6
RUS	Russian Federation	National	2015	8.0 [55]	162.29	194.33	248.96	421.68	2665.8
ESP	Spain	Subnational	1989–1990	19.0 [56]	157.49	263.78	234.63	525.39	1145.0
SWE	Sweden	Subnational	1997–2006	13.0 [57]	188.92	229.14	305.55	503.14	14,272.5
CHE	Switzerland	Subnational	1977–2005	11.8 [58]	188.47	137.66	308.20	350.49	8953.3
UKR	Ukraine	Subnational	1986–1997	11.0 [59]	149.60	114.19	248.40	261.74	1417.9
GBR	United Kingdom	Subnational	1998–2005	4.1 [60]	201.51	165.01	311.62	403.32	7937.9
USA	United States	Subnational	2007–2013	7.5 [61]	274.59	186.38	406.67	444.37	23,478.6

^a^ Each PUFA consumption is the average value of the consumption for the 20 years before the study period. ^b^ GDP per capita is the average value of the GDP per capita for the 20 years before the study period. PUFA—polyunsaturated fatty acid; AA—arachidonic acid; DHA—docosahexaenoic acid; LCPUFA—long-chain PUFA; and GDP—gross domestic product.

**Table 3:** The instruments of eight PUFAs and schizophrenia in MR analyses.

						Effect Size Estimates for PUFAs ^a^			Effect Size Estimates for Schizophrenia ^b^		
PUFA	SNP	Chr	Nearby Gene	Effect Allele	F Statistics	*β*	SE	*p*	ln (OR)	SE	*p*
Linoleic acid (LA, 18:2 ω-6)	rs10740118	10	*JMJD1C*	G	32.91	0.248	0.043	8.08 × 10^−9^	0.0093	0.0110	0.399
	rs174547	11	*FADS1*	C	1218.48	1.474	0.042	4.98 × 10^−274^	0.0205	0.0114	0.072
	rs16966952	16	*NTAN1, PDXDC1*	G	62.96	0.351	0.044	1.23 × 10^−15^	−0.0394	0.0117	0.001
γ-linolenic acid (GLA, 18:3 ω-6)	rs174547	11	*FADS1*	T	253.26	0.016	0.001	2.29 × 10^−72^	−0.0205	0.0114	0.072
	rs16966952	16	*NTAN1, PDXDC1*	G	36.81	0.006	0.001	5.0 × 10^−11^	−0.0394	0.0117	0.001
Dihomo-γ-linolenic acid (DGLA, 20:3 ω-6)	rs174547	11	*FADS1*	T	1211.88	−0.350	0.010	2.63 × 10^−151^	−0.0205	0.0114	0.072
	rs16966952	16	*NTAN1, PDXDC1*	G	119.70	0.220	0.020	7.55 × 10^−65^	−0.0394	0.0117	0.001
Arachidonic acid (AA, 20:4 ω-6)	rs174547	11	*FADS1*	T	4526.15	1.691	0.025	3.00 × 10^−971^	−0.0205	0.0114	0.072
	rs16966952	16	*NTAN1, PDXDC1*	G	40.77	0.199	0.031	2.43 × 10^−10^	−0.0394	0.0117	0.001
α-linolenic acid (ALA, 18:3 ω-3)	rs174547	11	*FADS1*	C	256.00	0.016	0.001	3.47 × 10^−64^	0.0205	0.0114	0.072
Eicosapentaenoic acid (EPA, 20:5ω3)	rs3798713	6	*ELOVL2*	C	49.00	0.035	0.005	1.93 × 10^−12^	0.0081	0.0110	0.459
	rs174538	11	*FADS1*	G	275.56	0.083	0.005	5.37 × 10^−58^	−0.0211	0.0117	0.072
Docosapentaenoic acid (DPA, 22:5 ω-3)	rs780094	2	*GCKR*	T	32.11	0.017	0.003	9.04 × 10^−9^	0.0007	0.0111	0.948
	rs3734398	6	*ELOVL2*	C	177.78	0.040	0.003	9.61 × 10^−44^	0.0088	0.0110	0.424
	rs174547	11	*FADS1*	T	625.00	0.075	0.003	3.79 × 10^−154^	−0.0205	0.0114	0.072
Docosahexaenoic acid (DHA, 22:6 ω-3)	rs2236212	6	*ELOVL2*	G	65.15	0.113	0.014	1.26 × 10^−15^	−0.0085	0.0110	0.439

^a^ Summary statistic for GWAS of PUFA from “Genome-wide association study of plasma N6 polyunsaturated fatty acids within the cohorts for heart and aging research in genomic epidemiology consortium” [23], “Genetic loci associated with plasma phospholipid *n*-3 fatty acids: a meta-analysis of genome-wide association studies from the CHARGE Consortium” [24]. ^b^ Summary statistics for GWAS of schizophrenia from “Biological insights from 108 schizophrenia-associated genetic loci” [29]. PUFA—polyunsaturated fatty acid; SNP—single-nucleotide polymorphism; Chr—chromosome; SE—standard error; and OR—odds ratio.

**Table 4:** The multivariable MR analyses for ω-6 and ω-3 PUFAs with risk for schizophrenia.

	Number of SNP Instruments (Related to PUFA Group) ^a^	Number of SNPs in Analysis	F Statistic	Result in IVW Method ^a,b^	
				OR (95% CI)	*p*
Arachidonic acid (AA, 20:4 ω-6)	3	8	85.33	0.981 (0.976–0.986)	0.009
Eicosapentaenoic acid (EPA, 20:5 ω-3)	6	8	30.67	1.359 (0.702–2.633)	0.090
Linoleic acid (LA, 18:2 ω-6)	3	8	6.46	1.005 (0.957–1.055)	0.805
α-linolenic acid (ALA, 18:3 ω-3)	6	8	6.02	3.747 (0.019–728.509)	0.512

^a^ Summary statistic for GWAS of PUFA from “Genome-wide association study of plasma N6 polyunsaturated fatty acids within the cohorts for heart and aging research in genomic epidemiology consortium” [23], “Genetic loci associated with plasma phospholipid *n*-3 fatty acids: a meta-analysis of genome-wide association studies from the CHARGE Consortium” [24]. ^b^ Summary statistics for GWAS of schizophrenia from “Biological insights from 108 schizophrenia-associated genetic loci” [29]. PUFA–polyunsaturated fatty acid; SNP–single-nucleotide polymorphism; Chr–chromosome; SE–standard error; and OR–odds ratio.

## Data Availability

Food consumption data were obtained from the Food and Agriculture Organization of the United Nations, and the nutrition information of food was obtained from the Australian Food Composition Database (previously called NUTTAB). GDP per capita in each country was obtained from the World Bank. The incidence rates of schizophrenia and PUFA related datasets used in Mendelian randomization analyses are from published studies, which are cited in the chapter “References”. The genetic statistical data of schizophrenia can be downloaded from the Psychiatry Genomics Consortium database.

## Supplementary Materials

The following supporting information can be downloaded at https://www.mdpi.com/article/10.3390/nu15051195/s1. Table S1. Food supply quantity (g per capita per day) in different country; Table S2. Characteristics of participants included in the PUFA meta-analysis GWAS; Table S3. Overall enrolled studies about incidence of schizophrenia in each country; Table S4. Enrolled studies about incidence of affective disorder and dietary PUFAs across countries; Table S5. Pleiotropy assessment of rs174547 in FDAS1 and rs16966952 in NTAN1/PDXDC1; Table S6. The instruments adjusted for the preceding fatty acid levels of three PUFAs and schizophrenia in MR analyses.; Figure S1. Results of linkage disequilibrium analysis between (A) rs174547 and rs10832027, rs1582763 in chromosome 11; (B) rs16966952 and rs10521222, rs1558902 in chromosome 16. Refs. [23,24,35,36,38,39,40,41,42,43,44,45,46,47,48,49,50,51,52,53,54,55,56,57,58,59,60,61,62,63,64,65,66,67,68,69,70,71,72,73,74,75,76,77,78,79,80,81,82] cited in Supplementary Materials.
