# Supplementary material for: Association between Arachidonic Acid and the Risk of Schizophrenia: A Cross-National Study and Mendelian Randomization Analysis
Source: Nutrients. 2023 Feb 27;15(5):1195. doi: 10.3390/nu15051195 (PMC10005211; doi:10.3390/nu15051195)
Supplement: Supplementary file 1 [file nutrients-15-01195-s001.zip › nutrients-2183769-Supplementary_materials/nutrients-2183769-Supplementary_TablesS2-6&FigureS1.docx]

**Supplementary Tables**

**Table S2. Characteristics of participants included in the PUFA meta-analysis GWAS.**

**Table S3. Overall enrolled studies about incidence of schizophrenia in each country.**

**Table S4. Enrolled studies about incidence of affective disorder and the dietary PUFAs across countries.**

**Table S5. Pleiotropy assessment of rs174547 in *FDAS1* and rs16966952 in *NTAN1/PDXDC1.***

**Table S6. The instruments adjusted for the preceding fatty acid levels of three PUFAs and schizophrenia in MR analyses.**

**Figure S1. Results of linkage disequilibrium analysis between (A) rs174547 and rs10832027, rs1582763 in in chromosome 11; (B) rs16966952 and rs10521222, rs1558902 in chromosome 16.**

**Table S2. Characteristics of participants included in the PUFA** **meta-analysis GWAS.**

| **ω-6 meta-analysis GWAS^a^, n=8631^*^** | | | | | | |
| --- | --- | --- | --- | --- | --- | --- |
|  |  |  | **PUFA concentration, % of total fatty acid** | | | |
| **Cohort studies** | **N** | **Women %** | **LA** | **GLA** | **DGLA** | **AA** |
| ARIC | 3269 | 51 | 21.97 | 0.11 | 3.33 | 11.45 |
| CARDIA | 1507 | 53 | 21.98 | 0.11 | 3.26 | 11.80 |
| CHS | 2404 | 62 | 19.96 | 0.09 | 3.13 | 10.87 |
| InCHIANTI | 1075 | 55 | 24.78 | NA | NA | 8.00 |
| MESA | 707 | 53 | 20.90 | 0.12 | 3.26 | 12.10 |
| **ω-3 meta-analysis GWAS^b^, n=8866** | | | | | | |
|  |  |  | **PUFA concentration, % of total fatty acid** | | | |
| **Cohort studies** | **N** | **Women %** | **ALA** | **EPA** | **DPA** | **DHA** |
| ARIC | 3268 | 51 | 0.14 | 0.56 | 0.90 | 2.82 |
| CARDIA | 1507 | 53 | 0.19 | 0.85 | 0.94 | 3.09 |
| CHS | 2326 | 61 | 0.15 | 0.59 | 0.83 | 2.97 |
| InCHIANTI | 1075 | 55 | 0.44 | 0.61 | NA | 2.29 |
| MESA | 690 | 53 | 0.18 | 0.88 | 0.95 | 3.66 |

Note: * Less than the total number of participants, as it described in original papers;

a Summary statistics for GWAS of ω-6 PUFA from “Genome-wide association study of plasma N6 polyunsaturated fatty acids within the cohorts for heart and aging research in genomic epidemiology consortium.”[23],

b Summary statistics for GWAS of ω-3 PUFA “Genetic loci associated with plasma phospholipid n-3 fatty acids: a meta-analysis of genome-wide association studies from the CHARGE Consortium.”[24].

ARIC, Atherosclerosis Risk in Communities Study; CHS, the Cardiovascular Health Study; CARDIA, the Coronary Artery Risk Development in Young Adults Study; InCHIANTI, the Invecchiare in Chianti Study; MESA, the Multi-Ethnic Study of Atherosclerosis (MESA); GWAS, Genome Wide Association Study; PUFA, polyunsaturated fatty acid; LA, Linoleic acid; GLA, γ-linolenic acid; DGLA, Dihomo-γ-linolenic acid; AA, Arachidonic acid; ALA, α-Linolenic acid; EPA, Eicosapentaenoic acid; DPA, Docosapentaenoic acid; DHA, Docosahexaenoic acid.

**Table S3. Overall enrolled studies about incidence of schizophrenia in each country**

| **Pub**  **Date** | **Country** | **Area** | **Case Find Method** | **Epoch** | **Duration** | **Diagnostic Method** | **Including**  **Criteria** | **Diagnostic Criteria** | **Diagnostic Criteria Narrow** | **Age Range** |
| --- | --- | --- | --- | --- | --- | --- | --- | --- | --- | --- |
| 2019 | Australia[62] | subnational | HosIn and Hosout | 1992-2015 | 24 | ChartDiag | First admission | ICD10, DSM | schizophrenia, schizoaffective disorder | 15+ |
| 2000 | Brazil[38] | subnational | HosIn | 1964-94 | 4 | ChartDiag | First admission | ICD8 | schizophrenia | Unkown |
| 1992 | Canada[63] | subnational | MultipleInstitutions | 1982-1986.5 | 2.5 | FaceToFace | First episode | ICD9, DSM3, RDC, Feighner, Other | schizophrenia | 15-54 |
| 1992 | Canada[64] | subnational | HosIn | 1983-1987 | 4 | Services | First episode | ICD9, DSM3 | 295.0, 297.0, 298.0, 301.22 | All |
| 2004 | Canada[39] | subnational | HosIn and Hosout | 1995-1998 | 4 | ChartDiag | First episode | ICD9 | 295 | 15-35 |
| 2021 | Chile[40] | national | HosIn and Hosout | 2004-2017 | 14 | ChartDiag | First episode | ICD10 | F20-29 | 10-65 |
| 1997 | Costa Rica[41] | national | HosIn | 1979-1981 | 3 | ChartDiag | First episode | ICD9 | 295.0 to 295.9 | Unkown |
| 1990 | Croatia[42] | national | MultipleInstitutions | 1965-1984 | 20 | ChartDiag | First admission | ICD7/8/9 | schizophrenia | 15+ |
| 2013 | Denmark[43] | national | MultipleInstitutions | 1995-2008 | 14 | ChartDiag | First episode | ICD10 | F20 | 15-64 |
| 1993 | Finland[65] | subnational | MultipleInstitutions | 1983-1988 | 5 | ChartDiag | First contact | ICD8, DSM3 | 295 | 15-59 |
| 2020 | Finland[66] | Finland | MultipleInstitutions | 1996-2014 | 18 | CaseNoteRevSys | First admission | ICD10 | F20-29 | Unkown |
| 2011 | Finland[44] | Finland | HosIn | 1980&2003 | 24 | ChartDiag | First admission | ICD8, ICD10 | 295, F20/25 | 15-64 |
| 1999 | Germany[45] | subnational | HosIn | 1987-1989 | 2 | FaceToFace | First admission | ICD9, CATEGO | 295/297/298.3/298.4 | 12-49 |
| 1977 | Iceland[46] | national | HosIn | 1967 | 2 | ChartDiag | First admission | ICD8 | 295 | All |
| 1993 | India[47] | subnational | Survey | 1987-1988 | 2 | Surveies | First episode | ICD9 | 295 | 18-64 |
| 1984 | Ireland[48] | national | HosIn | 1974-1977 | 3 | FaceToFace | First contact | ICD9 | schizophrenia | 15-64 |
| 1993 | Italy[67] | subnational | HosIn | 1982-1989 | 8 | ChartDiag | First contact | ICD9 | 295 | 15+ |
| 2000 | Italy[68] | national | HosIn | 1984-1994 | 10 | ChartDiag | First admission | ICD9 | 295 | All |
| 2017 | Italy[49] | subnational | HosIn | 2008-2011 | 4 | FaceToFace | First episode | ICD10 | F20-29, F30-33 | 18-65 |
| 2014 | Italy[69] | subnational | MultipleInstitutions | 2005-2007 | 3 | FaceToFace | First episode | ICD10 | F20-29 | 15-54 |
| 2012 | Italy[70] | subnational | MultipleInstitutions | 2002-2009 | 8 | ChartDiag | First episode | ICD10 | F10–F29 & F30–F33 | 18-64 |
| 1992 | Japan[50] | subnational | CommunitySurvey | 1979-1980 | 2 | FaceToFace | First episode | ICD9 | schizophrenia | 15-54 |
| 2000 | Netherlands[51] | subnational | HosIn | 1986-1997 | 11 | ChartDiag | First register | ICD9 | 295/297 | 15-64 |
| 2018 | New Zealand[52] | national | HosIn | 2014 | 1 | ChartDiag | First register | ICD10 | schizophrenia-related | 18–65 |
| 1985 | Norway[53] | national | HosInOut | 1982-1983 | 2 | ChartDiag | First admission | ICD8,9 | 295 | All |
| 2017 | Republic of Korea[71] | national | HosIn and Hosout | 2006-2007 | 1 | ChartDiag | First admission | ICD10 | F20 | Unkown |
| 2021 | Republic of Korea[54] | national | HosIn and Hosout | 2008-2017 | 12 | ChartDiag | First admission | ICD10 | F20-29 | Unkown |
| 2019 | Russian Feder-ation[55] | national | HosIn | 2005&2015 | 11 | ChartDiag | First admission | ICD10 | schizophrenia | Unkown |
| 1995 | Spain[56] | subnational | MultipleInstitutions | 1989-1990 | 2 | FaceToFace | First contact | ICD9, DSM3, CATEGO | schizophrenia | 15-54 |
| 2020 | Spain[72] | subnational | HosIn and Hosout | 2008-2015 | 8 | ChartDiag | First appearance | ICD9 | 295.XX | 15-34 |
| 2010 | Sweden[57] | subnational | HosIn and Hosout | 1997-2006 | 10 | ChartDiag | First register | ICD10 | F20 | 18-44 |
| 2001 | Sweden[73] | subnational | HosIn | 1978-1994 | 17 | ChartDiag | First admission | ICD8, ICD9 | 295 | 15-69 |
| 2019 | Switzerland[74] | subnational | CommunitySurvey | 2013-2017 | 5 | ChartDiag | First register | ICD11 | schizophrenia | Unkown |
| 2007 | Switzerland[58] | subnational | HosIn | 1977-2005 | 29 | ChartDiag | First admission | ICD8/9/10 | schizophrenia | All |
| 2000 | Ukraine[59] | subnational | HosIn | 1986-1997 | 12 | ChartDiag | First admission | ICD9 | 295 | All |
| 1997 | The United Kingdom[75] | subnational | MultipleInstitutions | 1978-1980; 1992-1994 | 14 | FaceToFace | First episode | ICD10 | F20 | 16-64 |
| 1994 | The United Kingdom[76] | subnational | HosIn and Hosout | 1975-86 | 12 | CaseNoteRevSys | First admission | ICD7 | 295 | 15-54 |
| 2020 | The United Kingdom[77] | subnational | HosIn and Hosout | 2004-2015 | 12 | ChartDiag | First diagnosis | ICD10 | schizophrenia-related | 15+ |
| 2010 | The United Kingdom[60] | subnational | MultipleInstitutions | 1998-2005 | 8 | ChartDiag | First episode | ICD10 | F20-F29 | 16+ |
| 2004 | The United Kingdom[78] | subnational | MultipleInstitutions | 1994-1996 | 3 | FaceToFace | First episode | ICD10 | F20-F29 | 16-64 |
| 2017 | The United States of America[61] | subnational | MultipleInstitutions, HspIn and HopOut | 2007-2013 | 7 | ChartDiag | First diagnosis | ICD9 | 295-297 | 15-59 |

**Table S4. Enrolled studies about incidence of affective disorder and the dietary PUFAs across countries.**

|  | **Denmark[79]** | **Finland[80]** | **Italy[69]** | **United.Kingdom[60]** | **Netherlands[81]** | **Sweden[82]** |
| --- | --- | --- | --- | --- | --- | --- |
| **Published Year** | 2016 | 2017 | 2014 | 2010 | 2003 | 2015 |
| **Area** | national | national | national | subnatioanl | national | national |
| **Case Find Method** | HosInOut, 'HosIn | HosInOut, 'HosIn | MultipleInstitutions | HosIn | HosIn | HosInOut |
| **Epoch** | 1995-2010 | 2011-2014 | 2005-2007 | 1998-2005 | 1990-1996 | 1991-2010 |
| **Duration** | 16 | 4 | 3 | 7 | 7 | 20 |
| **Diagnostic Criteria** | ICD10 | ICD10 | ICD10 | ICD-10 | ICD9 | ICD-9, ICD-10 |
| **Diagnostic Criteria_narrow** | F30, F31, F34.0 | F30-31 | F30-31 | F30–31.9 | 296.0, 296.2; 296.3; 296.4; 296.5 | ICD-9: 296.0, 296.4-296.8, 296A.296C-296E, 296; ICD-10: F30, F31 |
| **Including Criteria** | First register | First episode | First contact | First episode | First admission | First episode |
| **Age Range** | 4-65 | >15 | 15-64 | >15 | 15-54 | NA |
| **Incidence**  **Bipolar disorder** | 12.70 | 4.50 | 1.60 | 3.22 | 1.43 | 4.00 |
| **Incidence**  **Major depression disorder** | NA | NA | 2.20 | 5.41 | 2.06 | NA |
| **ALA (C18:3 ω-3)/g** | 0.20 | 0.14 | 0.21 | 0.26 | 0.21 | 0.19 |
| **DHA (C22:6 ω-3)/mg** | 23.70 | 20.27 | 19.89 | 22.15 | 17.07 | 12.67 |
| **Total ω-3 LCPUFA/mg** | 56.93 | 48.94 | 45.67 | 51.86 | 42.54 | 31.71 |
| **Total ω-3 PUFA/mg** | 258.49 | 188.40 | 253.47 | 306.91 | 250.42 | 217.04 |
| **LA (C18:2 ω-6)/g** | 1.81 | 0.90 | 2.28 | 2.22 | 1.57 | 2.06 |
| **AA (C20:4 ω-6)/mg** | 27.57 | 12.40 | 20.93 | 18.72 | 20.01 | 17.59 |
| **Total ω-6 LCPUFA/mg** | 47.80 | 20.94 | 33.65 | 31.82 | 33.55 | 29.79 |
| **Total ω-6 PUFA/mg** | 1858.96 | 923.38 | 2309.56 | 2254.07 | 1602.69 | 2084.83 |

ICD, International Classification of Diseases; PUFA, polyunsaturated fatty acid; AA, arachidonic acid; DHA, docosahexaenoic acid; LCPUFA, long chain PUFA; GDP, gross domestic product.

**Table S5. Pleiotropy assessment of rs174547 in *FDAS1* and rs16966952 in *NTAN1/PDXDC1***

| **snp** | **trait** | **pmid** | **ancestry** | **beta** | **se** | **p** | **direction** | **n** | **unit** | **dataset** |
| --- | --- | --- | --- | --- | --- | --- | --- | --- | --- | --- |
| rs174547 | Plasma omega 6 polyunsaturated fatty acid levels arachidonic acid | 24823311 | European | 1.69 | NA | 0 | + | - | unit decrease | NHGRI-EBI_GWAS_Catalog |
| rs174547 | Arachidonic acid 20:4n6 | 24823311 | European | -1.691 | 0.0253 | 3.30E-971 | - | 8631 | % | CHARGE_Arachidonic-acid_EUR_2014 |
| rs174547 | Linoleic acid 18:2n6 | 24823311 | European | 1.474 | 0.0417 | 4.98E-274 | + | 8631 | % | CHARGE_Linoleic-acid_EUR_2014 |
| rs174547 | Lipid metabolism phenotypes | 22286219 | European | NA | NA | 8.00E-262 | NA | - | - | NHGRI-EBI_GWAS_Catalog |
| rs174547 | Lipid metabolism | 22286219 | European | NA | NA | 8.00E-262 | NA | - | - | dbGaP |
| rs174547 | Metabolite levels | 20037589 | European | 36.3 | 1.273 | 7.00E-179 | + | - | % variance explained | NHGRI-EBI_GWAS_Catalog |
| rs174547 | Electrocardiography | 20037589 | European | NA | NA | 7.00E-179 | NA | - | - | dbGaP |
| rs174547 | Glycerophospholipid levels | 26068415 | European | -0.1652 | 0.00585 | 2.00E-175 | - | - | unit increase | NHGRI-EBI_GWAS_Catalog |
| rs174547 | Glycerophospholipid levels | 26068415 | European | -0.1408 | 0.00504 | 9.00E-172 | - | - | unit increase | NHGRI-EBI_GWAS_Catalog |
| rs174547 | DPA 22:5n3 | 21829377 | European | -0.0746 | 0.0028 | 3.79E-154 | - | 8866 | % | CHARGE_DPA_EUR_2011 |
| rs174547 | Plasma docosapentaenoic acid levels | 21829377 | European | NA | NA | 3.79E-154 | NA | 8866 | - | GRASP |
| rs174547 | Phospholipid levels plasma | 21829377 | European | -0.07 | 0.002647 | 4.00E-154 | - | - | % increase | NHGRI-EBI_GWAS_Catalog |
| rs174547 | Phospholipids | 21829377 | European | NA | NA | 4.00E-154 | NA | - | - | dbGaP |
| rs174547 | Dihomo-gamma-linolenic acid | 24823311 | European | 0.355 | 0.0136 | 2.63E-151 | + | 8631 | % | CHARGE_Dihomo-gamma-linolenic-acid_EUR_2014 |
| rs174547 | Glycerophospholipid levels | 26068415 | European | -0.1157 | 0.004581 | 9.00E-141 | - | - | unit increase | NHGRI-EBI_GWAS_Catalog |
| rs174547 | Adrenic acid 22:4n6 | 24823311 | European | -0.0483 | 0.0019 | 6.26E-140 | - | 8631 | % | CHARGE_Adrenic-acid_EUR_2014 |
| rs174547 | Metabolic traits | 21886157 | European | 0.178 | 0.007783 | 9.00E-116 | + | - | unit decrease | NHGRI-EBI_GWAS_Catalog |
| rs174547 | Metabolism | 21886157 | European | NA | NA | 9.00E-116 | NA | - | - | dbGaP |
| rs174547 | Glycerophospholipid levels | 26068415 | European | -0.0992 | 0.005108 | 5.00E-84 | - | - | unit increase | NHGRI-EBI_GWAS_Catalog |
| rs174547 | Plasma alpha linolenic acid levels | 21829377 | European | NA | NA | 7.00E-84 | NA | 8866 | - | GRASP |
| rs174547 | Plasma omega 6 polyunsaturated fatty acid levels gamma linolenic acid | 24823311 | European | -0.02 | 0.001111 | 2.00E-72 | - | - | unit increase | NHGRI-EBI_GWAS_Catalog |
| rs174547 | Gamma-linolenic acid 18:3n6 | 24823311 | European | -0.0156 | 9.00E-04 | 2.29E-72 | - | 8631 | % | CHARGE_Gamma-linolenic-acid_EUR_2014 |
| rs174547 | Glycerophospholipid levels | 26068415 | European | -0.0764 | 0.004306 | 2.00E-70 | - | - | unit increase | NHGRI-EBI_GWAS_Catalog |
| rs174547 | Phospholipid levels plasma | 21829377 | European | -0.02 | 0.00118 | 3.00E-64 | - | - | % decrease | NHGRI-EBI_GWAS_Catalog |
| rs174547 | Phospholipids | 21829377 | European | NA | NA | 3.00E-64 | NA | - | - | dbGaP |
| rs174547 | ALA 18:3n3 | 21829377 | European | 0.0159 | 9.00E-04 | 3.47E-64 | + | 8866 | % | CHARGE_ALA_EUR_2011 |
| rs174547 | Plasma eicosapentaenoic acid levels | 21829377 | European | NA | NA | 1.83E-57 | NA | 8866 | - | GRASP |
| rs174547 | EPA 20:5n3 | 21829377 | European | -0.082 | 0.0051 | 1.83E-57 | - | 8866 | % | CHARGE_EPA_EUR_2011 |
| rs174547 | Glycerophospholipid levels | 26068415 | European | -0.0663 | 0.00487 | 3.00E-42 | - | - | unit increase | NHGRI-EBI_GWAS_Catalog |
| rs174547 | Triglycerides | 24097068 | European | 0.0469 | 0.0035 | 1.04E-40 | + | 174696 | IVNT | GLGC_TG_EUR_2013 |
| rs174547 | Glycerophospholipid levels | 26068415 | European | -0.0884 | 0.00664 | 2.00E-40 | - | - | unit increase | NHGRI-EBI_GWAS_Catalog |
| rs174547 | Red cell distribution width | 27863252 | European | -0.04908 | 0.00371 | 6.77E-40 | - | 173480 | IVNT | Astle-W_Blood-Cell-Traits_EUR_2016 |
| rs174547 | Low density lipoprotein | 24097068 | European | -0.0505 | 0.0038 | 7.99E-38 | - | 170015 | IVNT | GLGC_LDL_EUR_2013 |
| rs174547 | Pulse rate | UKBB | European | 0.03383 | 0.00263 | 8.59E-38 | + | 317756 | IVNT | Neale-B_UKBB_EUR_2017 |
| rs174547 | Total cholesterol | 24097068 | European | -0.0472 | 0.0037 | 1.35E-35 | - | 184184 | IVNT | GLGC_TC_EUR_2013 |
| rs174547 | FADS1 gene expression in human liver | 19060906 | European | NA | NA | 5.00E-35 | NA | 19840 | - | GRASP |
| rs174547 | Oleic acid 18:1n9 | 23362303 | European | 0.2282 | 0.0194 | 5.57E-32 | + | 8961 | % | CHARGE_Oleic-acid_EUR_2013 |
| rs174547 | Plasma oleic acid | 23362303 | European | NA | NA | 5.57E-32 | NA | 8961 | - | GRASP |
| rs174547 | Age related disease endophenotypes | 27790247 | European | NA | NA | 1.00E-29 | NA | - | - | NHGRI-EBI_GWAS_Catalog |
| rs174547 | High density lipoprotein | 24097068 | European | -0.0389 | 0.0035 | 4.05E-27 | - | 184044 | IVNT | GLGC_HDL_EUR_2013 |
| rs174547 | Age related diseases mortality and associated endophenotypes | 27790247 | European | NA | NA | 7.00E-25 | NA | - | - | NHGRI-EBI_GWAS_Catalog |
| rs174547 | Mean platelet volume | 27863252 | European | -0.0389 | 0.00379 | 1.14E-24 | - | 173480 | IVNT | Astle-W_Blood-Cell-Traits_EUR_2016 |
| rs174547 | Triglycerides | 20686565 | Mixed | 0.0481 | 0.005 | 6.00E-23 | + | 96598 | Z-score | GLGC_TG_Mixed_2010 |
| rs174547 | Triglycerides | 20686565 | Mixed | NA | NA | 6.00E-23 | NA | 100184 | - | GRASP |
| rs174547 | Metabolite levels lipid measures | 27005778 | European | 0.4 | 0.04062 | 7.00E-23 | + | - | unit decrease | NHGRI-EBI_GWAS_Catalog |
| rs174547 | Platelet count | 27863252 | European | 0.03721 | 0.003824 | 2.21E-22 | + | 173480 | IVNT | Astle-W_Blood-Cell-Traits_EUR_2016 |
| rs174547 | HDL cholesterol | 20686565 | Mixed | NA | NA | 1.00E-21 | NA | 100184 | - | GRASP |
| rs174547 | High density lipoprotein | 20686565 | Mixed | -0.0477 | 0.0051 | 1.11E-21 | - | 99900 | Z-score | GLGC_HDL_Mixed_2010 |
| rs174547 | Total cholesterol | 20686565 | Mixed | NA | NA | 2.00E-20 | NA | 100184 | - | GRASP |
| rs174547 | Total cholesterol | 20686565 | Mixed | -0.0491 | 0.0053 | 2.29E-20 | - | 100184 | Z-score | GLGC_TC_Mixed_2010 |
| rs174547 | Red blood cell count | 27863252 | European | 0.03451 | 0.00373 | 2.29E-20 | + | 173480 | IVNT | Astle-W_Blood-Cell-Traits_EUR_2016 |
| rs174547 | Stearic acid 18:0 | 23362303 | European | -0.1773 | 0.0193 | 4.42E-20 | - | 8961 | % | CHARGE_Stearic-acid_EUR_2013 |
| rs174547 | Plasma stearic acid | 23362303 | European | NA | NA | 4.42E-20 | NA | 8961 | - | GRASP |
| rs174547 | LDL cholesterol | 20686565 | Mixed | NA | NA | 1.00E-19 | NA | 100184 | - | GRASP |
| rs174547 | Low density lipoprotein | 20686565 | Mixed | -0.0486 | 0.0054 | 1.17E-19 | - | 95454 | Z-score | GLGC_LDL_Mixed_2010 |
| rs174547 | Height | 25429064 | East Asian | -0.037 | 0.00429 | 6.00E-18 | - | - | unit increase | NHGRI-EBI_GWAS_Catalog |
| rs174547 | Fasting glucose | 22885924 | European | -0.019 | 0.0022 | 1.33E-17 | - | 133010 | mmol/l | MAGIC_FG-METABO_EUR_2012 |
| rs174547 | Plasma omega 6 polyunsaturated fatty acid levels linoleic acid | 26584805 | East Asian | -0.048 | 0.00578 | 1.00E-16 | - | - | unit decrease | NHGRI-EBI_GWAS_Catalog |
| rs174547 | Granulocyte percentage of myeloid white cells | 27863252 | European | -0.03025 | 0.00375 | 7.06E-16 | - | 173480 | IVNT | Astle-W_Blood-Cell-Traits_EUR_2016 |
| rs174547 | Triglycerides | 19060906 | European | NA | NA | 1.70E-15 | NA | 19840 | - | GRASP |
| rs174547 | Triglycerides | 19060906 | European | 0.06 | 0.007842 | 2.00E-14 | + | - | SD increase | NHGRI-EBI_GWAS_Catalog |
| rs174547 | Triglycerides | 19060906 | European | NA | NA | 2.00E-14 | NA | - | - | dbGaP |
| rs174547 | Monocyte percentage of white cells | 27863252 | European | 0.0283 | 0.003739 | 3.77E-14 | + | 173480 | IVNT | Astle-W_Blood-Cell-Traits_EUR_2016 |
| rs174547 | Trans fatty acid levels | 25646338 | Mixed | -0.0032 | 0.0004262 | 6.00E-14 | - | - | unit decrease | NHGRI-EBI_GWAS_Catalog |
| rs174547 | Cis trans-18:2 | 25646338 | European | 0.0032 | 4.00E-04 | 6.17E-14 | + | 8013 | % | CHARGE_Cis-trans-182_EUR_2015 |
| rs174547 | Hemoglobin concentration | 27863252 | European | 0.0275 | 0.003721 | 1.47E-13 | + | 173480 | IVNT | Astle-W_Blood-Cell-Traits_EUR_2016 |
| rs174547 | Pulse rate | UKBB | European | 0.03184 | 0.004469 | 1.06E-12 | + | 110153 | IVNT | Neale-B_UKBB_EUR_2017 |
| rs174547 | HDL cholesterol | 19060906 | European | NA | NA | 2.00E-12 | NA | 19840 | - | GRASP |
| rs174547 | HDL cholesterol | 21347282 | African | NA | NA | 2.00E-12 | NA | 8090 | - | GRASP |
| rs174547 | HDL cholesterol | 19060906 | European | 0.09 | 0.01279 | 2.00E-12 | + | - | SD decrease | NHGRI-EBI_GWAS_Catalog |
| rs174547 | Cholesterol hdl | 19060906 | European | NA | NA | 2.00E-12 | NA | - | - | dbGaP |
| rs174547 | Plasma palmitoleic acid | 23362303 | European | NA | NA | 2.97E-12 | NA | 8961 | - | GRASP |
| rs174547 | Palmitoleic acid 16:1n7 | 23362303 | European | 0.023 | 0.0033 | 2.97E-12 | + | 8961 | % | CHARGE_Palmitoleic-acid_EUR_2013 |
| rs174547 | Sphingolipid levels | 26068415 | European | -0.026 | 0.003765 | 5.00E-12 | - | - | unit increase | NHGRI-EBI_GWAS_Catalog |
| rs174547 | Height | UKBB | European | -0.0125 | 0.001815 | 5.63E-12 | - | 336474 | IVNT | Neale-B_UKBB_EUR_2017 |
| rs174547 | Granulocyte count | 27863252 | European | -0.02593 | 0.003767 | 5.82E-12 | - | 173480 | IVNT | Astle-W_Blood-Cell-Traits_EUR_2016 |
| rs174547 | Sum neutrophil eosinophil counts | 27863252 | European | -0.02578 | 0.003761 | 7.15E-12 | - | 173480 | IVNT | Astle-W_Blood-Cell-Traits_EUR_2016 |
| rs174547 | Sphingolipid levels | 26068415 | European | -0.0295 | 0.004508 | 6.00E-11 | - | - | unit increase | NHGRI-EBI_GWAS_Catalog |
| rs174547 | Hematocrit | 27863252 | European | 0.02423 | 0.003705 | 6.16E-11 | + | 173480 | IVNT | Astle-W_Blood-Cell-Traits_EUR_2016 |
| rs174547 | Self-reported asthma | UKBB | European | -0.00534 | 0.0008199 | 6.86E-11 | - | 337159 | risk diff | Neale-B_UKBB_EUR_2017 |
| rs174547 | Nap during day | UKBB | European | 0.009819 | 0.001514 | 8.91E-11 | + | 337074 | - | Neale-B_UKBB_EUR_2017 |
| rs174547 | Eosinophil count | 27863252 | European | -0.02407 | 0.003741 | 1.24E-10 | - | 173480 | IVNT | Astle-W_Blood-Cell-Traits_EUR_2016 |
| rs174547 | Mean corpuscular volume | 27863252 | European | -0.02382 | 0.003704 | 1.26E-10 | - | 173480 | IVNT | Astle-W_Blood-Cell-Traits_EUR_2016 |
| rs174547 | Height | 28146470 | Mixed | -0.016 | 0.0026 | 2.60E-10 | - | 458927 | IVNT | GIANT_Height_Mixed_2017 |
| rs174547 | Sum basophil neutrophil counts | 27863252 | European | -0.02375 | 0.003765 | 2.81E-10 | - | 173480 | IVNT | Astle-W_Blood-Cell-Traits_EUR_2016 |
| rs174547 | Neutrophil count | 27863252 | European | -0.02351 | 0.003758 | 3.94E-10 | - | 173480 | IVNT | Astle-W_Blood-Cell-Traits_EUR_2016 |
| rs174547 | Myeloid white cell count | 27863252 | European | -0.02348 | 0.003778 | 5.15E-10 | - | 173480 | IVNT | Astle-W_Blood-Cell-Traits_EUR_2016 |
| rs174547 | Asthma | UKBB | European | -0.00503 | 0.0008184 | 7.77E-10 | - | 336782 | risk diff | Neale-B_UKBB_EUR_2017 |
| rs174547 | White blood cell count | 27863252 | European | -0.0231 | 0.003761 | 8.16E-10 | - | 173480 | IVNT | Astle-W_Blood-Cell-Traits_EUR_2016 |
| rs174547 | RR interval | 20639392 | European | NA | NA | 8.20E-10 | NA | 38991 | - | GRASP |
| rs174547 | Sum eosinophil basophil counts | 27863252 | European | -0.02255 | 0.003745 | 1.74E-09 | - | 173480 | IVNT | Astle-W_Blood-Cell-Traits_EUR_2016 |
| rs174547 | Resting heart rate | 20639392 | European | 6.2 | 1.034 | 2.00E-09 | + | - | m SD increase | NHGRI-EBI_GWAS_Catalog |
| rs174547 | Heart rate | 20639392 | European | NA | NA | 2.00E-09 | NA | - | - | dbGaP |
| rs174547 | Heart rate | 23583979 | Mixed | 0.3278 | 0.0538 | 6.99E-09 | + | 89275 | bpm | HRGene_HR_Mixed_2013 |
| rs174547 | Plateletcrit | 27863252 | European | 0.02208 | 0.003837 | 8.64E-09 | + | 173480 | IVNT | Astle-W_Blood-Cell-Traits_EUR_2016 |
| rs174547 | Hair or balding pattern: pattern 4 | UKBB | European | -0.00783 | 0.001462 | 8.58E-08 | - | 154988 | risk diff | Neale-B_UKBB_EUR_2017 |
| rs16966952 | Dihomo-gamma-linolenic acid | 2.48E+07 | European | -0.2204 | 0.013 | 7.55E-65 | - | 8631 | % | CHARGE_Dihomo-gamma-linolenic-acid_EUR_2014 |
| rs16966952 | Linoleic acid 18:2n6 | 2.48E+07 | European | 0.3512 | 0.0439 | 1.23E-15 | + | 8631 | % | CHARGE_Linoleic-acid_EUR_2014 |
| rs16966952 | Sitting height | UKBB | European | -0.01451 | 0.002056 | 1.71E-12 | - | 336172 | IVNT | Neale-B_UKBB_EUR_2017 |
| rs16966952 | Whole body water mass | UKBB | European | -0.01128 | 0.001685 | 2.13E-11 | - | 331315 | IVNT | Neale-B_UKBB_EUR_2017 |
| rs16966952 | Whole body fat-free mass | UKBB | European | -0.01113 | 0.001682 | 3.67E-11 | - | 331291 | IVNT | Neale-B_UKBB_EUR_2017 |
| rs16966952 | Gamma-linolenic acid 18:3n6 | 2.48E+07 | European | -0.0061 | 9.00E-04 | 5.05E-11 | - | 8631 | % | CHARGE_Gamma-linolenic-acid_EUR_2014 |
| rs16966952 | Trunk fat-free mass | UKBB | European | -0.01096 | 0.001676 | 6.15E-11 | - | 331030 | IVNT | Neale-B_UKBB_EUR_2017 |
| rs16966952 | Trunk predicted mass | UKBB | European | -0.0109 | 0.00167 | 6.78E-11 | - | 330995 | IVNT | Neale-B_UKBB_EUR_2017 |
| rs16966952 | Basal metabolic rate | UKBB | European | -0.01142 | 0.001764 | 9.52E-11 | - | 331307 | IVNT | Neale-B_UKBB_EUR_2017 |
| rs16966952 | Height | UKBB | European | -0.01203 | 0.00189 | 2.00E-10 | - | 336474 | IVNT | Neale-B_UKBB_EUR_2017 |
| rs16966952 | Leg fat-free mass left | UKBB | European | -0.0111 | 0.001752 | 2.35E-10 | - | 331258 | IVNT | Neale-B_UKBB_EUR_2017 |
| rs16966952 | Arachidonic acid 20:4n6 | 2.48E+07 | European | -0.1989 | 0.0314 | 2.43E-10 | - | 8631 | % | CHARGE_Arachidonic-acid_EUR_2014 |
| rs16966952 | Leg predicted mass left | UKBB | European | -0.01099 | 0.00174 | 2.62E-10 | - | 331253 | IVNT | Neale-B_UKBB_EUR_2017 |
| rs16966952 | Leg fat-free mass right | UKBB | European | -0.01103 | 0.001751 | 2.98E-10 | - | 331285 | IVNT | Neale-B_UKBB_EUR_2017 |
| rs16966952 | Leg predicted mass right | UKBB | European | -0.0109 | 0.001739 | 3.72E-10 | - | 331285 | IVNT | Neale-B_UKBB_EUR_2017 |
| rs16966952 | Arm fat-free mass right | UKBB | European | -0.00982 | 0.001658 | 3.13E-09 | - | 331221 | IVNT | Neale-B_UKBB_EUR_2017 |
| rs16966952 | Arm predicted mass right | UKBB | European | -0.00972 | 0.001652 | 3.94E-09 | - | 331216 | IVNT | Neale-B_UKBB_EUR_2017 |

Note: Data from PhenoScanner ([www.phenoscanner.medschl.cam.ac.uk/](http://www.phenoscanner.medschl.cam.ac.uk/)),

|  | | | | | | **Effect size estimates for PUFAs ^a^** | | | **Effect size estimates for schizophrenia ^b^** | | | **Resluts in IVW method** | |
| --- | --- | --- | --- | --- | --- | --- | --- | --- | --- | --- | --- | --- | --- |
| **PUFA** | **SNP** | **Chr** | **Nearby gene** | **Effect allele** | **F Stat** | ***β*** | **SE** | **p** | ***ln(OR)*** | **SE** | **p** | **OR (95% CI)** | **p** |
| γ-linolenic acid  (GLA, 18:3 ω-6) | rs174547 | 11 | *FADS1* | T | 88.24 | 0.0085 | 0.0009 | 1.47*10^-20^ | -0.0205 | 0.0114 | 0.072 | 0.240  (0.006-0.094) | 0.002 |
|  | rs16966952 | 16 | *NTAN1, PDXDC1* | G | 21.54 | 0.0042 | 0.0009 | 3.05*10^-06^ | -0.0394 | 0.0117 | 0.001 |  |  |
| Dihomo-γ-linolenic acid  (DGLA, 20:3 ω-6) | rs174547 | 11 | *FADS1* | T | 826.85 | -0.4192 | 0.0145 | 1.74*10^-148^ | -0.0205 | 0.0114 | 0.072 | 1.005  (0.953-1.060) | 0.845 |
|  | rs16966952 | 16 | *NTAN1, PDXDC1* | G | 246.62 | 0.2021 | 0.0128 | 1.79*10^-56^ | -0.0394 | 0.0117 | 0.001 |  |  |
| Arachidonic acid  (AA, 20:4 ω-6) | rs174547 | 11 | *FADS1* | T | 3848.69 | 1.7901 | 0.0287 | 5.00*10^-850^ | -0.0205 | 0.0114 | 0.072 | 0.986  (0.984-0.988) | 0.021 |
|  | rs16966952 | 16 | *NTAN1, PDXDC1* | G | 72.09 | 0.2911 | 0.0341 | 1.26*10^-17^ | -0.0394 | 0.0117 | 0.001 |  |  |

**Table S6. The instruments adjusted for the preceding fatty acid of three PUFAs and schizophrenia in MR analyses.**

PUFA, polyunsaturated fatty acid; SNP, single-nucleotide polymorphism; Chr, chromosome; SE, standard error; OR, odds ratio; 95% CI, 95% confidence interval; IVW, inverse-variance weighted.

a Summary PUFAs statistics of analysis adjusted for each PUFA precursor from “Genome-wide association study of plasma N6 polyunsaturated fatty acids within the cohorts for heart and aging research in genomic epidemiology consortium.” [23].

b Summary statistics for schizophrenia from “Biological insights from 108 schizophrenia-associated genetic loci” [24].


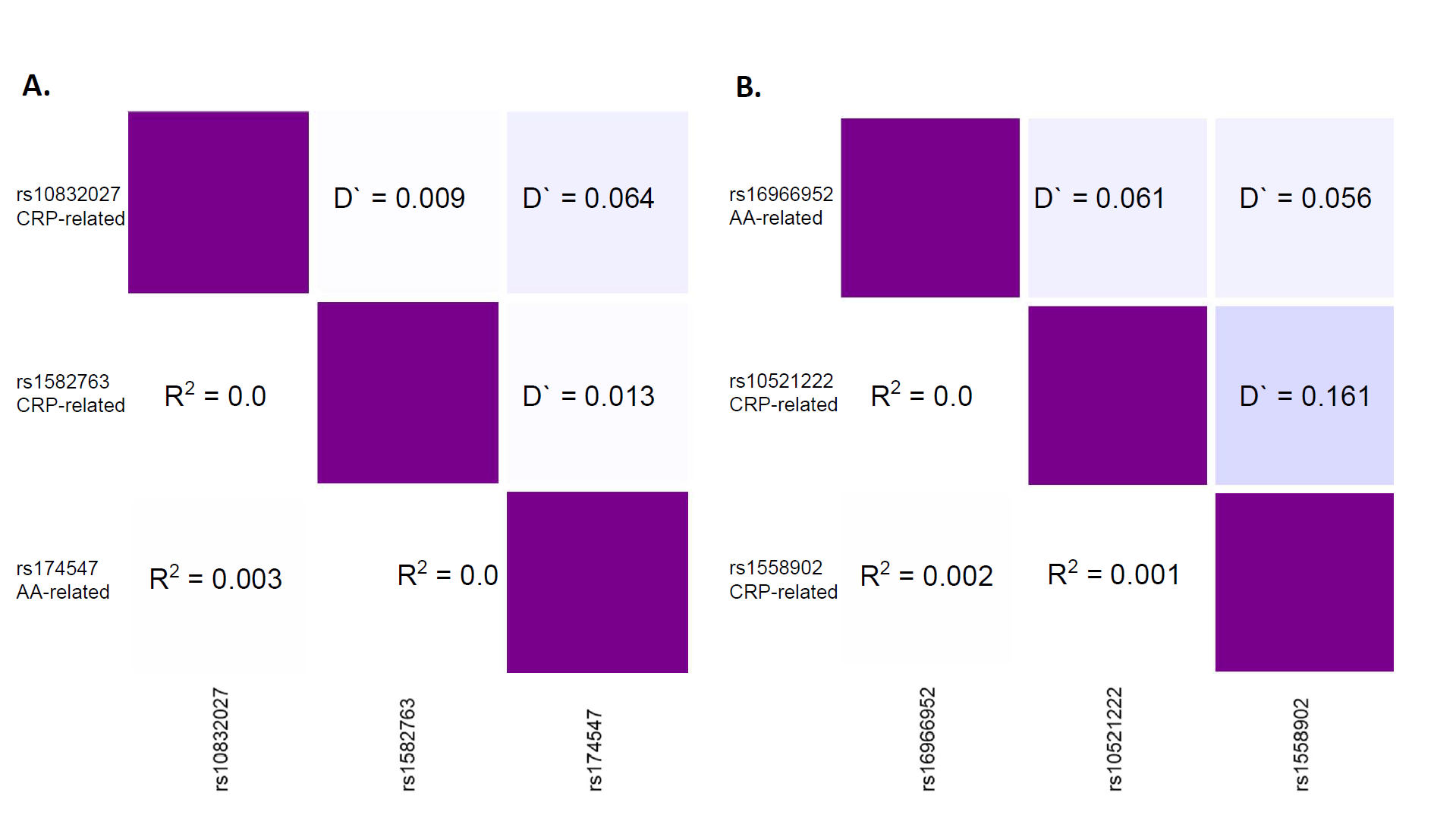


**Figure S1. Results of linkage disequilibrium analysis between (A) rs174547 and rs10832027, rs1582763 in in chromosome 11; (B) rs16966952 and rs10521222, rs1558902 in chromosome 16.** rs16966952 and rs174547 are significant SNPs in GWAS of AA [23]; rs10521222, rs1558902, rs10832027 and rs1582763 are significant SNPs in GWAS of CRP [36]. CRP, C-reactive protein; AA, arachidonic acid. Visualization via LDlink [35].
